# Type‐II Dirac Fermions in Monolayer In_2_O: Interplay of Magnetotransport, Spin Hall Effect, and Superconductivity

**DOI:** 10.1002/advs.202524346

**Published:** 2026-04-13

**Authors:** Qing‐Bo Liu, De‐Ming Feng, Wen‐Neng Zhao, Yu‐Yang Zhao, Lun Xiong, Xing‐Yi Tan, Hua‐Hua Fu

**Affiliations:** ^1^ Hubei Key Laboratory of Optical Information and Pattern Recognition, School of Optical Information and Energy Engineering, School of Mathematics and Physics Wuhan Institute of Technology Wuhan China; ^2^ Department of Physics Chongqing Three Gorges University Wanzhou China; ^3^ College of Intelligent Systems Science and Engineering Hubei Minzu University Enshi China; ^4^ School of Physics and Wuhan National High Magnetic Field Center Huazhong University of Science and Technology Wuhan People's Republic of China; ^5^ Institute for Quantum Science and Engineering Huazhong University of Science and Technology Wuhan Hubei China

**Keywords:** magnetotransport, monolayer In_2_O, spin hall effect, superconductivity, topological states

## Abstract

Topological quasiparticle excited states, magnetotransport, spin Hall effect, and superconductivity in solid‐state materials have consistently been the four key issues in condensed matter physics. In this work, we theoretically demonstrate that monolayer ${\mathrm{In}}_{2}O$ provides an effective platform to explore these intertwined phenomena through its unique electronic topology. First‐principles calculations reveal a type‐II Dirac point near the Fermi level of the electronic band structure of monolayer ${\mathrm{In}}_{2}O$, which is split into two pairs of Weyl points with topological charges of ±1 in the presence of spin–orbit coupling. Robust edge states along the (100) direction confirm its topologically nontrivial nature. Remarkably, the system exhibits negative magnetoresistance below the temperature of 30 K with a significant Hall conductance and a predicted superconducting transition at 1.5 K, which are induced both by phonon softening and van Hove singularities. These theoretical findings establish the monolayer ${\mathrm{In}}_{2}O$ as a prototypical two‐dimensional material for investigating type‐II Dirac physics and the interplay of topological states, magnetotransport, and superconductivity.

## Graphical abstract

First‐principles calculations reveal that monolayer ${\mathrm{In}}_{2}O$ hosts type‐II Dirac fermions near the Fermi level, which split into Weyl points under spin‐orbit coupling. The material exhibits negative and giant magnetoresistance, a pronounced spin Hall effect, and phonon‐mediated superconductivity at 1.5 K, establishing it as a unique platform for exploring intertwined topological and transport phenomena in two dimensions.

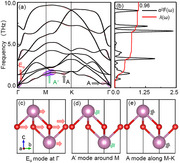

## 1 Introduction

Two‐dimensional (2D) materials have attracted significant attention in condensed matter physics and material science, due to their unique electronic [1, 2], magnetic [3], topological [4], superconducting [5], and phononic properties [6]. In particular, these characteristics make these materials exceptionally promising for applications in devices such as spintronics [7], single‐spin‐state quantum dots [8], and skyrmions [9]. For example, the topologically protected one‐dimensional edge states of 2D topological materials inherently exhibit robustness against undesired backscattering [10]. Furthermore, these materials, characterized by unique geometric structures, can be easily fabricated into various heterojunction structures in experiments, allowing the excitation of a wide range of highly tunable physical properties [11]. A notable example is the HgTe/CdTe and InAs/GaSb/AlSb quantum well structures, which exhibit the inspiring quantum spin Hall effect [12]; another exemplary case is the intrinsic monolayer $M_{4}\mathrm{XY}$ 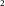 (M = Pd, Zr; X = S, Se, Te; and Y = Cl, Br, I) 2D materials, where superconductivity and magnetism coexist well [13]. Furthermore, sandwich‐structured TMD‐based 2D materials have been observed to exhibit novel charge density waves [14], dual layer superconductivity [15], and topological superconducting states [16, 17]. Their special three‐atom stacking structure, endowed with thickness, provides the necessary stability for the emergence of these unique physical phenomena. Clearly, the design and discovery of ideal 2D topological materials possessing multiple properties, including magnetism and superconductivity, along with topological features, is becoming increasingly crucial.

Topological insulators (TIs) [18], Dirac semi‐metals [19], Weyl semimetals [20, 21], nodal‐line semi‐metals [22], and superconductors [23, 24], have been predicted and subsequently observed in various solid‐state materials, accompanied by some extraordinary electronic transport phenomena. For example, a weak TI sample ${\mathrm{ZrTe}}_{5}$ demonstrates unsaturated magnetoresistance [25, 26] and resistivity anomalies, notably Hall resistivity with inversion. A Weyl semimetallic sample, ${\mathrm{Co}}_{3}\mathrm{Sn}$ 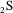 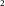, exhibits unusual magnetotransport behavior [27]. In addition, another class of superconductivity, namely chiral topological superconductivity, has been observed in the atomic insulator/ferromagnetic insulator heterostructure based on superconductor‐blocked materials (${\mathrm{Nb}}_{3}\mathrm{Br}$ 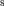/${\mathrm{NbSe}}_{2}$/${\mathrm{CrI}}_{3}$) [28]. Furthermore, topological superconductivity has been reported in a range of topological materials, such as Janus‐MoSH monolayers [29], biphenylene monolayers [30], monolayers of β‐${\mathrm{Bi}}_{2}\mathrm{Pd}$ [31], and 2D Janus transition metal sulfhydrates [32]. However, it is unfortunate that to date there have been no reports on the coexistence of magnetotransport, spin Hall effects, and superconductivity within the same 2D material.

In this work, we employ first‐principles calculations to systematically investigate the electronic structure of 2D ${\mathrm{In}}_{2}O$ monolayer [33]. Our findings reveal that the electronic band structure of this 2D material can form ideal type‐II Dirac points (DPs), accompanied by magnetoresistance (MR) phenomena and unusual spin Hall resistivity behavior. Notably, at temperatures below 1.5 K, it exhibits perfect superconducting behavior. Our research further indicates that, in the absence of spin–orbit coupling (SOC), this type‐II DP exists near the Fermi level of ${\mathrm{In}}_{2}O$. However, after introducing SOC, this DP splits to form two pairs of type‐II Weyl points (WPs) with topological charges of ±1. Moreover, ideal edge states can be observed along the (100) direction, both with and without SOC, confirming its nontrivial topological nature. Furthermore, our calculations show that at 30 K, the MR and spin Hall resistivity of ${\mathrm{In}}_{2}O$ exhibit a negative magnetoresistance (NMR) effect, a giant magnetoresistance (GMR) effect, and a large Hall conductivity. Furthermore, the superconductivity of this material stems from its phonon acoustic branches, phonon softening, and van Hove singularities (vHSs). Our theoretical accomplishments not only offer an ideal material platform for studying type‐II Dirac points but also provide a perfect material specimen for exploring magneto‐transport, spin Hall effect, superconductivity, and their coexistence in 2D material systems.

## 2 Results and Discussion

### 2.1 Symmetry Analysis

To elucidate the topologically nontrivial states in layer group 71 where the monolayer ${\mathrm{In}}_{2}O$ exists, we should first prove the existence of DPs at the high‐symmetry path M–K in the first Brillouin zone (BZ) by using a two‐band k·p model as described by

(1) $$H_{kp}\left(k\right)=g_{x}\left(k\right)\sigma_{x}+g_{y}\left(k\right)\sigma_{y}+g_{z}\left(k\right)\sigma_{z},$$

where $k=(k_{x},k_{y})$, $\sigma_{x,y,z}$ represents the three Pauli matrices, and $g_{x,y,z}\left(k\right)$ represents the complex functions versus $k_{x}$ and $k_{z}$.

Then, we further consider a twofold rotation symmetry 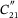 (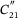:(x,y)>(x−y, $\bar{y}$)), the combinatorial symmetry of the time‐reversal symmetry T and the space‐inversion symmetry P: T P along the high‐symmetry path M‐K in layer group 71. Under 2D irreps: $\Gamma_{1}$ $\Gamma_{2}$ [34, 35] of the wave group $G_{2}^{1}$, the representation matrices of 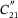 and T P can be written as


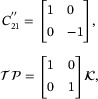

where K is a complex conjugate operator. Then, the two‐band k·p‐invariant Hamiltonian along the M‐K path is derived as

(2) $$\begin{array}{c} H_{kp}=\left[\begin{array}{cc} 0 & mk_{y}+nik_{x}\\ mk_{y}-nik_{x} & 0 \end{array}\right], \end{array}$$

where the parameters m and n are real constant coefficients, and i is an imaginary unit. According to this relation, a linear DP should be generated along the K–M path in this layer group.

When further taking into account the influence of SOC, a four‐band k·p‐invariant Hamiltonian should be adopted along the M–K path under a 4D irrep, that is, $\Gamma_{3}$ $\Gamma_{4}$ $\Gamma_{3}$ $\Gamma_{4}$ of the wave group $G_{4}^{1}$ [34, 35]. As a consequence, the related four‐band k·p model Hamiltonian can be derived as

$$\begin{array}{ccc} & & \;\;\;\;\;H_{kp}=\\ & & \left[\begin{array}{cccc} 0 & mk_{y}+nik_{x} & 0 & 0\\ mk_{y}-nik_{x} & 0 & 0 & 0\\ 0 & 0 & 0 & -mk_{y}-nik_{x}\\ 0 & 0 & -mk_{y}+nik_{x} & 0 \end{array}\right]. \end{array}$$

It should be noted that a two‐band k·p model can be effectively used to prove the existence of DPs at the high‐symmetry point K in layer group 71. The point K includes several fundamental symmetries, including 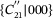 and $\left\{C_{3}^{+}|000\right\}$, and T P. Under 2D irreps: $\Gamma_{3}$ [34, 35] of the point group $D_{3}$, the representation matrices of symmetries 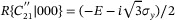, $R\left\{C_{3}^{+}|000\right\}=\sigma_{z}$ and RTP=−E, where i is an imaginary unit and E is an identity matrix. Then the two‐band k·p‐invariant Hamiltonian at K can be derived as

(3) $$\begin{array}{c} H_{kp}=C_{1}k_{x}\sigma_{z}+C_{2}k_{y}\sigma_{x}, \end{array}$$

where the parameters $C_{1}$ and $C_{2}$ are two real constant coefficients.

### 2.2 Material Example and Topological Properties

**FIGURE 1:**
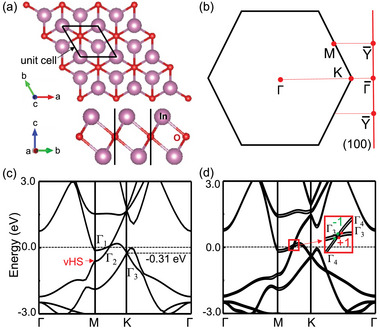
(a) Top and side views of the monolayer lattices of the 2D ${\mathrm{In}}_{2}O$, where the grep (red) atoms stand for In (O) and the black rhombus stands for the primitive cell. (b) The 2D BZ of the monolayer ${\mathrm{In}}_{2}O$, where the red line stands for the 1D BZ of ${\mathrm{In}}_{2}O$ along the (100) direction. (c) The band structure of the monolayer ${\mathrm{In}}_{2}O$ without SOC, here we can observe the ideal type‐II DPs near Fermi‐level protected by the 2D irreps: $B_{1}B$ 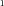. (d) The electronic band structure of the monolayer ${\mathrm{In}}_{2}O$ with SOC, where we can find that the ideal type‐II DPs can split off two WPs with the topological charge of +1 and two WPs with the topological charge of −1.

The crystal structure of the material sample ${\mathrm{In}}_{2}O$ in layer group 71 is illustrated in Figure 1a, where two In atoms (grep) and one O atom (red) are included in a unit cell, and the corresponding primitive lattice vectors are also given. The 2D Brillouin zone (BZ) is plotted in Figure 1b, where the red line represents the 1D BZ, formed by the projection of the 2D BZ along the (100) direction. The optimized lattice constants of ${\mathrm{In}}_{2}O$ are a=b= 3.25 Å, as described in Figure 1a, where the Wyckoff positions are 2d (0.333, 0.667, 0.4316) for the In atom and 1b (0, 0, 0.5) for the O atom. We find that the monolayer ${\mathrm{In}}_{2}O$ is composed of three atomic layers, with two layers of In atoms sandwiching one layer of O atoms in a sandwich structure. Each oxygen atom is surrounded by six In atoms, and each indium atom is surrounded by three oxygen atoms, bonded by strong covalent bonds. The thickness of each layer of ${\mathrm{In}}_{2}O$ is approximately 3.429 Å.

We first illustrate the electronic band structure of ${\mathrm{In}}_{2}O$ 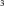 in the presence and absence of SOC in Figure 1c,d. As predicted from our previous symmetry analysis and the low‐energy k·p model, a type‐II DP emerges along the high‐symmetry path M–K in the electronic bands, situated precisely near the Fermi level. The two bands comprising this DP are derived from the 2D irreps $\Gamma_{1}$ $\Gamma_{2}$. Furthermore, our calculations reveal that this Type‐II DP is primarily contributed by the s and p orbitals of the In atoms within the material (see Figure S1). We also find that it can form vHSs at the point M in Figure 1c, which is associated with a density‐of‐states peak at 1.05 eV for this point as provided in Figure S1, presenting the possibility of superconductivity [36]. The band crossing at the K point forms the type‐I DP near −0.31 eV in Figure 1c, which is protected by 2D irreps: $\Gamma_{3}$. When SOC is taken into account, the type‐II DP splits into a pair of WPs characterized by the topological charges of ±1, as evidenced by the enlarged inset in Figure 1d, thus confirming the existence of DP and the topologically nontrivial nature of the material sample.

**FIGURE 2:**
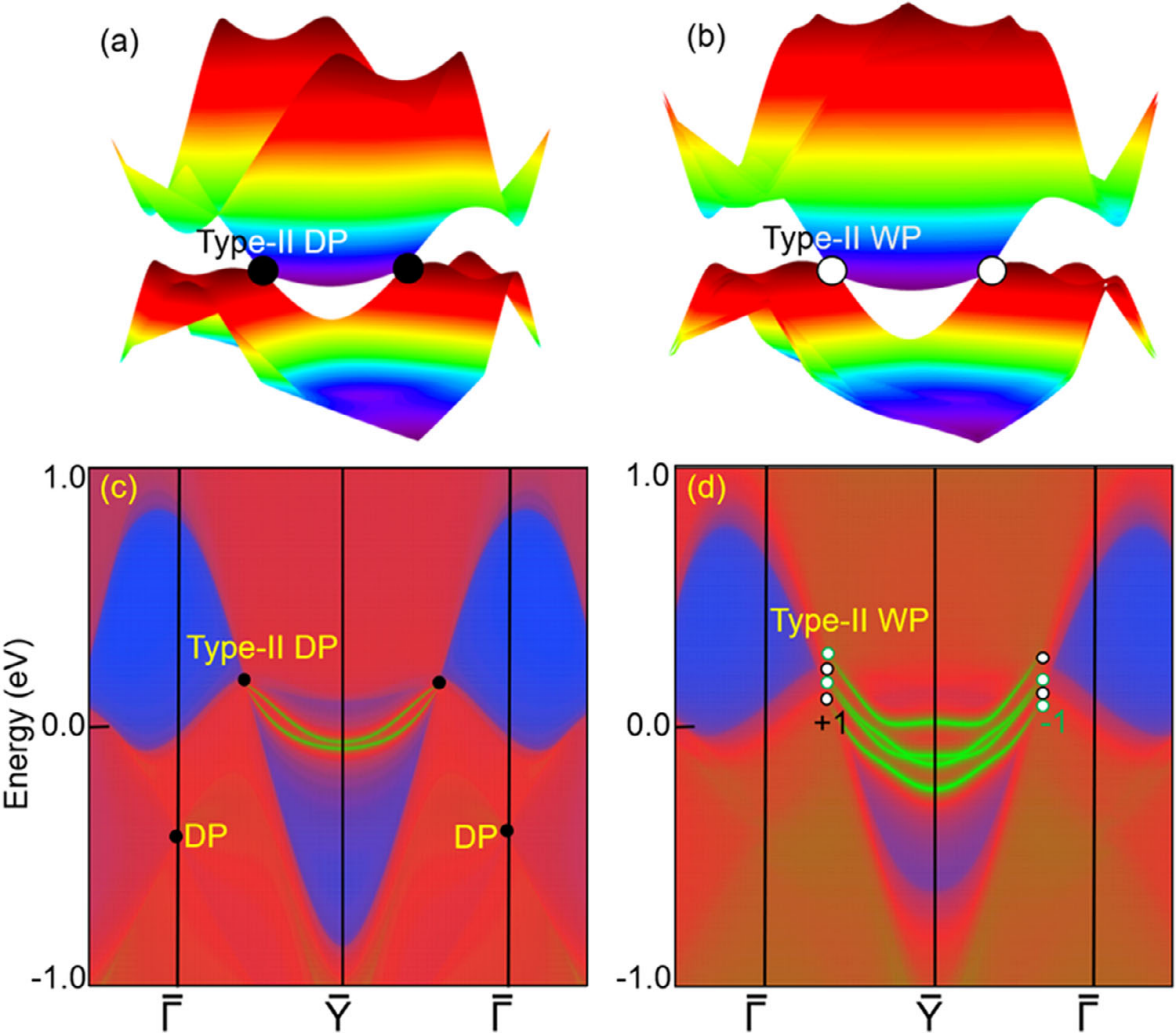
(a) The 3D electronic spectrum of type‐II DP near Fermi level without SOC. (b) The 3D electronic spectra of type‐II WP near Fermi level with SOC. (c) The edge state in the monolayer ${\mathrm{In}}_{2}O$ along the (100) direction without SOC. (d) The edge state in ${\mathrm{In}}_{2}O$ with SOC.

To further elucidate the topological characteristics of the ${\mathrm{In}}_{2}O$ monolayer, we performed calculations of the related three‐dimensional (3D) electronic band structure, considering both the presence and absence of SOC, as depicted in Figure 2a,b. These calculations also vividly illustrate the momentum–space distribution of a type‐II DP, and then split into a pair of WPs in both scenarios. As illustrated in Figure 1b, the high‐symmetry points Γ and K (M) are projected onto the $\bar{\Gamma }$ ($\bar{Y}$) point on the (100) surface, and the DP is projected along the path $\bar{Y}$‐$\bar{\Gamma }$ within the 1D BZ. In particular, we observe the formation of distinct surface arc states along the path $\bar{Y}$‐$\bar{\Gamma }$‐$\bar{Y}$ in the absence of SOC, as illustrated in Figure 2c. However, as the SOC is taken into account, four branches linking the projected WPs clearly exhibit arc‐shaped surface states, as shown in Figure 2d, indicating that the nontrivial surface arcs originally excited by the splitting of DP, are also generated accordingly. These observations robustly confirm the existence of nontrivial quasiparticle excited states in the monolayer ${\mathrm{In}}_{2}O$.

### 2.3 Magnetotransport Behavior and Spin Hall Effect

Upon examination of the electronic band structure of the monolayer ${\mathrm{In}}_{2}O$ illustrated in Figure 1c,d, we observe that this material may exhibit strong SOC. Moreover, it possesses intrinsic time‐reversal symmetry, which leads to an asymmetric distribution of spin splitting in momentum space. These fundamental properties endow the monolayer ${\mathrm{In}}_{2}O$ with a unique magnetotransport behavior and the spin Hall effect. Furthermore, the presence of nontrivial energy bands and pairs of WPs near the Fermi level significantly influences its magnetotransport features. Concurrently, the spin‐momentum locking characteristic of these nontrivial bands near the Fermi level, combined with the dimensionality effect of the material, ensures the occurrence of the spin Hall effect. Additionally, the Berry curvature at the nontrivial band crossings near the Fermi level further enhances the spin Hall effect. Based on these preliminary analyses of the band structure, the monolayer ${\mathrm{In}}_{2}O$ may emerge as a promising material candidate to exhibit both the novel magnetotransport phenomenon and the spin Hall effect.

**FIGURE 3:**
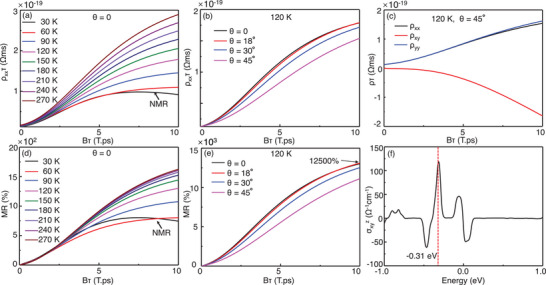
(a) The field‐dependent longitudinal resistivity with the temperatures increasing from 30 to 270 K under the external magnetic field along the z‐direction. (b) The field‐dependent longitudinal resistivity with the different angles between the magnetic field and the z‐axis under 120 K. (c) The field‐dependent longitudinal resistivity $\rho_{xx}$, $\rho_{yy}$ and transverse resistivity $\rho_{xy}$ under 120 K and the angle of 45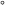. (d) The field‐dependent MR of $\rho_{xx}$ at the temperatures increasing from 30 to 270 K under the magnetic field along the z‐direction. (e) The field‐dependent MR of $\rho_{xx}$ with different angles between the magnetic field and the z‐axis at the temperature 120 K. (f) The spin Hall conductivity $\rho_{xy}^{z}$ versus the potential energy.

In order to verify the aforementioned predictions, we turn to the construction of an isotropic two‐band model based on four fundamental parameters, that is, electron and hole densities (denoted as $n_{e}$ and $n_{h}$, respectively), along with the corresponding mobilities ($\mu_{e}$ and $\mu_{h}$). Within this framework, the longitudinal resistivity $\rho_{xx}$ and the transverse resistivity $\rho_{yx}$ can be formulated as [25]

(4) $$\begin{array}{c} \rho_{xx}=\frac{1}{e}\frac{\left(n_{e}\mu_{e}+n_{h}\mu_{h}\right)+\left(n_{e}\mu_{h}+n_{h}\mu_{e}\right)\mu_{e}\mu_{h}B^{2}}{{\left(n_{e}\mu_{e}+n_{h}\mu_{h}\right)}^{2}+{\left(n_{h}-n_{e}\right)}^{2}\mu_{e}^{2}\mu_{h}^{2}B^{2}}, \end{array}$$

(5) $$\begin{array}{c} \rho_{yx}=\frac{B}{e}\frac{\left(n_{h}\mu_{h}^{2}-n_{e}\mu_{e}^{2}\right)+\left(n_{h}-n_{e}\right)\mu_{e}^{2}\mu_{h}^{2}B^{2}}{{\left(n_{e}\mu_{e}+n_{h}\mu_{h}\right)}^{2}+{\left(n_{h}-n_{e}\right)}^{2}\mu_{e}^{2}\mu_{h}^{2}B^{2}}, \end{array}$$

where we take some specific convention such that the positive value of $\rho_{yx}$ (B→∞), which indicates that a net hole concentration as $n_{h}>n_{e}$ is achieved. The calculation formula for magnetoresistance (MR) [37] is usually expressed as

(6) $$\begin{array}{c} \text{MR}=\frac{R(B)-R(0)}{R(0)}\times 100\% \end{array}$$

where R(B) responds to the resistance under an external magnetic field, and R(0) is the resistance at zero magnetic field. The spin Hall conductivity $\sigma_{xy}^{z}$ is evaluated via the Kubo formula in the linear response scheme, with the following form to calculate the related this specific conductivity [38]

(7) $$\begin{array}{c} \sigma_{xy}^{z}=-\frac{e^{2}}{\hbar }\frac{1}{VN_{k}}\sum_{n}\sum_{k}f_{nk}\Omega_{xy,n}^{z}\left(k\right) \end{array}$$

(8) $$\begin{array}{c} \Omega_{xy,n}^{z}\left(k\right)=\hbar \sum_{m\neq n}\frac{-2\;\text{Im}\;\langle nk|{\hat{j}}_{x}^{z}\left|mk\rangle \langle mk\right|{\hat{v}}_{y}\left|nk\right\rangle }{{\left(E_{nk}-E_{mk}\right)}^{2}} \end{array}$$

where $f_{nk}$ is the Fermi–Dirac distribution function, n is the electronic band index, $E_{nk}$ is the energy eigenvalue, V is the volume of primitive cell, and $N_{k}$ is the number of k point. In the first BZ, the spin current operator is defined as ${\hat{j}}_{i}^{z}=\frac{1}{2}\left\{{\hat{s}}_{z},{\hat{v}}_{i}\right\}$, where ${\hat{s}}_{z}=\frac{\hbar }{2}{\hat{\sigma }}_{z}$ is the spin matrix, and ${\hat{v}}_{i}=\frac{1}{\hbar }\frac{\partial H(k)}{\partial k_{i}}$ is the velocity operator. It is noted that $\sigma_{xy}^{z}$ describes the generation of a spin current along the x‐direction when the external electric field is applied along the y‐direction, while the polarization of the spin current occurs along the z‐direction. Following these relations, we may obtain the MR, the Hall resistivity, and the corresponding spin Hall conductivity, as illustrated in Figure 3.

First, we observe that when the angle θ between the external magnetic field and the z‐axis is set to $0^{\circ }$, the longitudinal resistivity $\rho_{xx}$ undergoes a dramatic change with increasing temperature, as depicted in Figure 3a. From 30 to 270 K, it exhibits nonlinear characteristics. Intriguingly, in the low temperature range below 30 K, $\rho_{xx}$ demonstrates a pronounced negative magnetoresistance (NMR) effect. Furthermore, at 120 K, we adjust the angle θ of the magnetic field to finite values such as $18^{\circ }$, $30^{\circ }$, and $45^{\circ }$ relative to the z‐axis. Figure 3b presents the variation of $\rho_{xx}$ with the external magnetic field at these angles. For comparison purposes, the variation curve of $\rho_{xx}$ at θ=$0^{\circ }$ is also included. We find that the Hall resistivity $\rho_{xx}$ decreases significantly as θ increases. To further elucidate the fundamental physics of Hall resistivity, we also calculate three representative Hall resistivity components, namely $\rho_{xx}$, $\rho_{xy}$, and $\rho_{yy}$, at 120 K and θ=$45^{\circ }$, as illustrated in Figure 3c. These numerical results indicate that, under low magnetic fields, $\rho_{xx}$ and $\rho_{yy}$ are nearly equal, while $\rho_{xy}$ is significantly different from the previous two Hall resistivity components.

**FIGURE 4:**
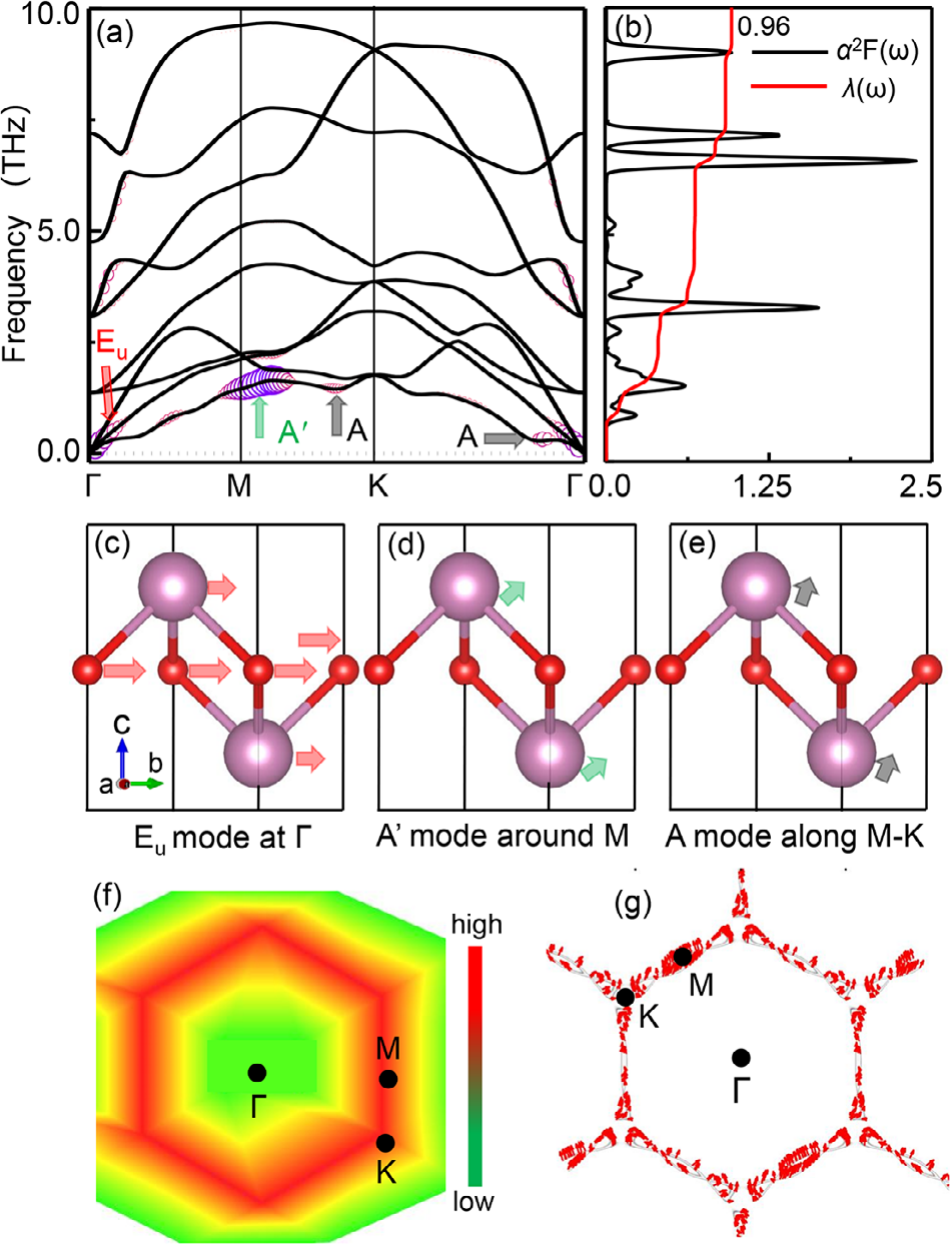
Phonon and superconducting features of ${\mathrm{In}}_{2}O$. (a) and (b) Phonon dispersion and the phonon dispersions weighted by the magnitude of the EPC, the Eliashberg spectral function $\alpha^{2}F\left(\omega \right)$, and the integrated strength of EPC λ(ω). (c) The phonon vibration mode of the lowest phonon band at Γ point. (d) The mode of vibration of the phonon of the lowest phonon band at point M. (e) The mode of vibration of the lowest phonon‐softed band along the M–K and Γ–K paths. (f) The momentum‐resolved EPC $\lambda_{q\nu }$ within the Fermi level. (g) The spin textures within the Fermi level.

To further validate the Hall resistivity features discussed above, we calculated the magnetoresistance (MR) of monolayer ${\mathrm{In}}_{2}O$ under various temperatures and different angles θ with respect to the external magnetic field. The corresponding results are presented in Figure 3d,e. When the angle θ is fixed at $0^{\circ }$, the MR exhibits a pronounced magnetic‐field dependence as the temperature increases from 30 to 270 K. As shown in Figure 4d, this evolution closely resembles the field‐dependent behavior of the Hall conductivity displayed in Figure 3a. Moreover, the MR shows an approximately linear dependence on the magnetic field and remains unsaturated under relatively weak magnetic fields. At low temperatures (e.g., 30 K), a pronounced negative magnetoresistance (NMR) emerges when the magnetic field is increased to higher values. This behavior can be understood in terms of intrinsic two‐dimensional quantum transport mechanisms. Specifically, quantum interference between time‐reversed electron trajectories enhances backscattering at zero magnetic field, giving rise to weak localization. The application of a magnetic field breaks time‐reversal symmetry and suppresses this interference, leading to an increase in conductivity and consequently negative magnetoresistance. In systems containing In atoms, spin–orbit coupling is generally strong and can induce weak antilocalization behavior in two dimensions. However, under finite magnetic fields, Zeeman splitting can disrupt spin coherence, partially suppress the quantum interference correction, and result in an effective negative magnetoresistance over a certain field range. In addition, an external magnetic field can induce carrier spin polarization, suppress spin‐flip scattering processes, and enhance carrier mobility, further contributing to the observed decrease in resistance with increasing magnetic field.

In particular, at a temperature of 120 K, the magnetic resistance reaches 1300%, indicating the emergence of the GMR phenomenon. Our calculations further demonstrate that below 120 K, the material can exhibit both unsaturated MR and a GMR effect (extending up to 12500%), even when the angle θ is adjusted to some others, such as $18^{\circ }$, $30^{\circ }$, and $45^{\circ }$. It is noted that the external magnetic field breaks the time‐reversal symmetry, thereby modulating the Berry curvature in the electronic band structure. This modification can reshape the properties of the edge states, stabilize topologically protected dissipationless transport channels, and ultimately lead to a giant GMR. Furthermore, Figure 3f shows the variation in the spin Hall conductivity of the monolayer ${\mathrm{In}}_{2}O$ with respect to its chemical potential energy. It is apparent that the spin Hall conductivity reaches approximately 50 Ω/cm near its Fermi level, which is generated by the type‐II WPs along the high‐symmetry path M–K in Figure 1d. Moreover, the material exhibits a maximum spin Hall conductivity value of approximately 125 Ω/cm in the region below its Fermi level (about −0.31 eV). We found that the Berry curvature generated by type‐I Weyl points is significantly stronger than that produced by type‐II Weyl points in Figure S3, resulting in the spin Hall conductance reaching its maximum at −0.31 eV. Furthermore, the DP associated with the point K at that specific energy level facilitates the emergence of this unique conduction phenomenon. To explore the tunability of the spin Hall effect in monolayer ${\mathrm{In}}_{2}O$ by external fields, we applied a perpendicular gate electric field of 0.5 eV/Å along the out‐of‐plane direction and found that it breaks the time‐reversal symmetry of the system, leading to the destruction of both type‐II and type‐I Dirac points in Figure S4a, while the spin Hall conductance is dramatically enhanced from a maximum value of 125 Ω/cm in the pristine case to 329.6 Ω/cm under the applied field Figure S4b, demonstrating that the spin Hall conductivity of monolayer ${\mathrm{In}}_{2}O$ can be effectively tuned by a vertical electric field, which offers a promising route for electrical control of spintronic devices based on this material.

### 2.4 Superconductivity of the Monolayer ${\mathrm{In}}_{2}O$

To gain a deeper understanding of the unconventional carrier transport phenomena observed in monolayer ${\mathrm{In}}_{2}O$, we will undertake an in‐depth analysis of the potentially nontrivial characteristics displayed by the phonon spectrum of this material. To accomplish this, we employ the phonon module within the Quantum ESPRESSO (QE) [39, 40, 41] software package, utilizing a 24×24×1 q‐grid in momentum space and applying the density functional perturbation theory (DFPT) [42] to compute its phonon dispersion spectrum, as depicted in Figure 4a. In particular, the absence of imaginary frequencies in the phonon spectrum and the absence of structural damage in molecular dynamics simulations under the temperature of 300 K (Figure S2) underscore the structural stability of the material sample. Moreover, the mode‐resolved magnitude of the electron–phonon coupling $\lambda_{q\nu }$ [43, 44] is calculated using the formula provided below

(9) $$\begin{array}{c} \lambda_{q\nu }=\frac{\gamma_{q\nu }}{\pi \hbar N\left(E_{F}\right)\omega_{q\nu }^{2}}, \end{array}$$

where $\gamma_{q\nu }$, $\omega_{q\nu }$, and $N(E_{F}$) stand for the phonon linewidth, the phonon frequency, and the electronic DOS at the Fermi level. The term $\gamma_{q\nu }$ is given as

$$\begin{array}{c} \gamma_{q\nu }=\frac{2\pi \omega_{q\nu }}{\Omega_{BZ}}\sum_{k,n,m}{\left|g_{kn,k+qm}^{\nu }\right|}^{2}\delta \left(\varepsilon_{kn}-\varepsilon_{F}\right)\delta \left(\varepsilon_{k+qm}-\varepsilon_{F}\right) \end{array}$$

where $\Omega_{BZ}$ stands for the volume of the first BZ, $\varepsilon_{kn}\left(\varepsilon_{k+qm}\right)$ stands for the Kohn–Sham eigenvalues, and $g_{kn,k+qm}^{\nu }$ is relative to the elements of the electron–phonon coupling (EPC) matrix [43]. Additionally, the Eliashberg electron–phonon spectral function $\alpha^{2}F\left(\omega \right)$ can be calculated by

$$\begin{array}{c} \alpha^{2}F\left(\omega \right)=\frac{1}{2\pi N(E_{F})}\sum_{q_{v}}\frac{\gamma_{q_{v}}}{\omega_{q_{v}}}\delta \left(\omega -\omega_{q_{v}}\right). \end{array}$$

The total EPC constant λ can be calculated using two distinct approaches, that is, (i) the BZ integration method: Integrating the mode‐resolved EPC constant $\gamma_{q_{v}}$ across the entire Brillouin zone while accounting for all phonon modes (ν) and (ii), the integration method of spectral function, that is, to integrate the Eliashberg spectral function $\alpha^{2}F\left(\omega \right)$ [45] as described

$$\begin{array}{c} \lambda \left(\omega \right)=\sum_{q_{v}}\lambda_{qv}=2\int_{0}^{\omega }\alpha^{2}F\left(\omega \right)d\omega . \end{array}$$

Furthermore, the superconducting transition temperature $T_{c}$ is calculated using the modified Allen–Dynes equation [46], which incorporates the EPC constant λ and the Coulomb pseudopotential $\mu^{*}$ as

(10) $$\begin{array}{c} T_{c}=\frac{\omega_{\log}}{1.2}\exp\left[-\frac{1.04(1+\lambda )}{\lambda -\mu^{*}\left(1+0.62\lambda \right)}\right], \end{array}$$

where $\omega_{\log}$ is the logarithmic average frequency

(11) $$\begin{array}{c} \omega_{\log}=\exp\left[\frac{2}{\lambda }\int_{0}^{\infty }\frac{d\omega }{\omega }\alpha^{2}F\left(\omega \right)\log\omega \right], \end{array}$$

and $\omega_{2}$ is the mean‐square frequency

(12) $$\begin{array}{c} \omega_{2}=\sqrt{\frac{1}{\lambda }\int_{0}^{\omega_{\max}}\left[\frac{2\alpha^{2}F\left(\omega \right)}{\omega }\right]\omega^{2}\;d\omega }. \end{array}$$

To maintain generality, we will use the widely accepted potential energy value of $\mu^{*}=0.10$ eV to evaluate the $T_{c}$ of this monolayer ${\mathrm{In}}_{2}O$. The computed results for the Eliashberg function $\alpha^{2}F\left(\omega \right)$ and the accumulated EPC coefficient λ(ω) are shown in Figure 4b. By examining the EPC parameter $\lambda_{q\nu }$, the Eliashberg spectral functions $\alpha^{2}F\left(\omega \right)$, and the frequency‐dependent coupling λ(ω), it is evident that the EPC constant λ is primarily contributed by the phonon modes near 1.25 THz at the M point (indicated by the green arrow) and the acoustic branch of the point Γ (indicated by the red arrow) in Figure 4a. Furthermore, when μ∗=0.10 and λ=0.96, using the Allen‐Dynes modified McMillian equation, the $T_{C}$ of the monolayer ${\mathrm{In}}_{2}O$ is estimated to be approximately 1.5 K. While this value is considerably lower than that of interface‐enhanced high‐temperature systems such as monolayer FeSe on ${\mathrm{SrTiO}}_{3}$ ($T_{C}$ > 65 K) [47], it is comparable to, or slightly higher than, many well‐established conventional 2D superconductors. For instance, it is similar to the $T_{C}$ of monolayer ${\mathrm{NbSe}}_{2}$ (1–3 K) [48], which is suppressed from its bulk value due to phase fluctuations, and falls within the lower range of gated transition metal dichalcogenides such as ${\mathrm{MoS}}_{2}$ (up to 10 K) [49]. Interestingly, the predicted $T_{C}$ is also remarkably close to that of magic‐angle twisted bilayer graphene (approximately 1.7 K) [50], despite the absence of moiré engineering in the ${\mathrm{In}}_{2}O$ system. Compared to elemental 2D superconductors like ultrathin Pb or In films ($T_{C}$ ranging from 1 K to above 5 K) [51], monolayer ${\mathrm{In}}_{2}O$ offers the distinct advantage of being a crystalline binary oxide—a relatively rare class of 2D superconductors. Its $T_{C}$ is well within the reach of standard dilution refrigeration, making it an experimentally accessible candidate for future synthesis and transport studies, and providing a new platform for exploring superconductivity in oxide‐based low‐dimensional systems.

It should be noted that the softening of the phonons with the phonon mode A [52] along the high‐symmetry paths K‐M and Γ‐K (depicted by the black arrow) in Figure 4a suggests an enhancement in its superconductivity. Not only that, we also discovered that the vHSs that appear in the band structure depicted in Figure S1 can markedly intensify the DOS at that specific location, a characteristic that is also conducive to the enhancement of $T_{C}$. Furthermore, the relevant phonon vibration modes surrounding the high‐symmetry points M and Γ with the phonon modes $E_{u}$ and $A^{\prime }$ are illustrated in Figure 4c,d. The strong electron–phonon coupling of these modes, combined with their out‐of‐plane vibrational character that enhances coupling to the Fermi level, is indicative of enhanced superconductivity. It is also clear that the O and In atoms exhibit significant vibrations along the xy‐plane, and in the phonon mode at the point Γ in Figure 4c, the amplitude of the O atoms is considerably higher than that of the In atoms.

It should be noted that, due to the robust O‐In bonds, O and In atoms exhibit a tendency to vibrate along the xy‐plane at 0 THz. However, at the point M, only the In atom vibrates along the yz‐plane, while the oxygen atom barely vibrates in the phonon mode, as depicted in Figure 4d. Consequently, the in‐plane vibration modes of In and O atoms couple with the charged electronic states surrounding the Fermi surface, leading to the occurrence of a high EPC constant. We calculate the momentum‐resolved EPC $\lambda_{q\nu }$ and spin textures within the Fermi level in Figure 4f,g. We find that $\lambda_{q\nu }$ exhibits significant enhancement along the *M*–*K* path, and the region of EPC enhancement coincides with the electronic state with strong spin orbit coupling characteristics in k‐space. The degeneracy of electronic states induced by spin–orbit coupling enhances the electron–phonon matrix element, resulting in localized strong EPC. And the softening of the phonons along the *M*–*K* path can also enhance EPC in Figure 4f,g.

## 3 Conclusions

In summary, by combining first‐principles calculations with symmetry analysis, we conducted a systematic investigation of the topological characteristics, NMR, GMR, and superconducting phase transition of monolayer ${\mathrm{In}}_{2}O$. Our theoretical calculations revealed that, in the absence of SOC, the electronic band structure of the material sample exhibits ideal type‐II DPs near its Fermi level. Upon considering SOC, these DPs are further split into two pairs of type‐II WPs with topological charge of ±1. Notably, robust edge states are observed along the (100) direction with and without SOC, affirming the topological nontrivial nature of these states. Moreover, our theoretical research unveiled intriguing magnetotransport behaviors in this 2D material, encompassing NMR, GMR, and significant Hall conductance. Furthermore, we predict that, driven by the coupling between acoustic phonon modes, phonon softening, and vHSs, the monolayer ${\mathrm{In}}_{2}O$ exhibits phonon‐mediated superconductivity at a critical temperature $T_{C}$ of 1.5 K. These theoretical discoveries verify that the monolayer ${\mathrm{In}}_{2}O$ serves as an exemplary platform for exploring type II Dirac/Weyl states, MR and NMR transport phenomena, spin Hall effect, and low‐dimensional superconductivity. Furthermore, our research findings have enriched our understanding of topological physics and associated unconventional transport phenomena in 2D materials, offering effective pathways for experimental research and applications in the fields of nanoelectronics and low‐dimensional quantum device applications.

## 4 Experimental Section

The electronic bands and the phononic dispersion of ${\mathrm{In}}_{2}O$ are calculated using the density function theory (DFT) based on the projector‐augmented wave method and the Perdew–Burke–Ernzerhof (PBE) functional within the Vienna ab initio Simulation Package (VASP) [53, 54]. The energy cut‐off for the plane‐wave basis set is set at 520 eV. The integration in the first BZ is done using the Monkhorst‐Pack k point mesh with a grid spacing below 0.02 Å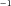, resulting in a k‐mesh size of 9×9×1. The convergence criteria were established at 1$\times 10^{-6}$ eV for energy and 0.01 eV for forces. A vacuum region of approximately 15 Å is included to prevent mirror interactions between periodic layers. The edge states are analyzed using the open‐source WANNIERTOOLS [55] code based on a Wannier tight‐binding (TB) model constructed with WANNIER90 [56]. In the magneto‐conductivity calculation, we use a k‐mesh of 201×201×201 and set the broadening width of the Fermi‐Dirac distribution function to 200 meV. The number of slices in the sequence of electronic motion trajectory points (${\mathrm{Nslice}}_{-}\mathrm{BTau}$ 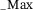) is set at 20 000. The electronic band irreps are copulated using the IRVSP [57] program on the electronic Hamiltonian of the TB model.

## Conflicts of Interest

The authors declare no competing financial interest.

## Supporting information


**Supporting File**: advs75194‐sup‐0001‐SuppMat.pdf.

## Data Availability

The data that support the findings of this study are available from the corresponding author upon reasonable request.

## References

[1] K. S. Novoselov , A. Mishchenko , A. Carvalho , and A. H. Castro Neto , “2D Materials and van der Waals Heterostructures,” Science 353 (2016): aac9439. 27471306 10.1126/science.aac9439

[2] Q. H. Wang , K. Kalantar‐Zadeh , A. Kis , J. N. Coleman , and M. S. Strano , “Electronics and Optoelectronics of Two‐Dimensional Transition Metal Dichalcogenides,” Nature Nanotechnology 7 (2012): 699–712. 10.1038/nnano.2012.193 23132225

[3] X. Xu , W. Yao , D. Xiao , and T. F. Heinz , “Spin and Pseudospins in Layered Transition Metal Dichalcogenides,” Science 346 (2014): 1254–1257.

[4] Y. Saito , T. Nojima , and Y. Iwasa , “Superconductivity Protected by spin‐Valley Locking in Ion‐Gated ${\mathrm{MoS}}_{2}$ ,” Nature Physics 12 (2016): 144–149.

[5] B. Huang , G. Clark , E. Navarro‐Moratalla , et al., “Layer‐Dependent Ferromagnetism in a van der Waals Crystal Down to the Monolayer limit,” Nature 546 (2017): 270–273. 28593970 10.1038/nature22391

[6] Q.‐B. Liu , Z.‐D. Guo , F.‐F. Du , et al., “The Type‐I, III Nodal Ring, Type‐I, III Quadratic Nodal Point, and Dirac Valley Phonons in 2D Kagome Lattices $M_{2}C$ (M = As, Bi, Cd, Hg, P, Sb, Zn),” Journal of Physics Condensed Matter 36 (2024): 325703. 10.1088/1361-648X/ad4430 38670080

[7] A. Avsar , H. Ochoa , F. Guinea , B. Özyilmaz , I. J. Vera‐Marun , and B. J. van Wees , “ *Colloquium*: Spintronics in Graphene and Other Two‐Dimensional Materials,” Reviews of Modern Physics 92 (2020): 021003.

[8] A. Splendiani , L. Sun , Y. Zhang , et al., “Emerging Photoluminescence in Monolayer ${\mathrm{MoS}}_{2}$ ,” Nano Letter 10 (2010): 1271–1275. 10.1021/nl903868w 20229981

[9] Y. Ji , S. Yang , H.‐B. Ahn , et al., “Direct Observation of Room‐Temperature Magnetic Skyrmion Motion Driven by Ultra‐Low Current Density in Van Der Waals Ferromagnets,” Advanced Materials 36 (2024): 2312013. 10.1002/adma.202312013 38270245

[10] Y. Ran , Y. Zhang , and A. Vishwanath , “One‐Dimensional Topologically Protected Modes in Topological Insulators With Lattice Dislocations,” Nature Physics 5 (2009): 298–303.

[11] X. Chen , Z. Zhou , B. Deng , et al., “Electrically Tunable Physical Properties of Two‐Dimensional Materials,” Nano Today 27 (2019): 99–119.

[12] P. C. Klipstein , “Erratum: Structure of the Quantum Spin Hall States in HgTe/CdTe and InAs/GaSb/AlSb Quantum Wells [Phys. Rev. B 91, 035310 (2015)],” Physical Review B 93 (2016): 199905.

[13] C. Ding , Q. Lu , D. Shao , et al., “Two‐Dimensional M‐Chalcogene Family With Tunable Superconducting, Topological, and Magnetic Properties,” Nano Letter 24 (2024): 9953–9960. 10.1021/acs.nanolett.4c02508 39102284

[14] T. Valla , A. V. Fedorov , P. D. Johnson , et al., “Quasiparticle Spectra, Charge‐Density Waves, Superconductivity, and Electron‐Phonon Coupling in 2H–${\mathrm{NbSe}}_{2}$ ,” Physical Review Letters 92 (2004): 086401. 14995798 10.1103/PhysRevLett.92.086401

[15] L. Yan , C. Ding , M. Li , et al., “Modulating Charge‐Density Wave Order and Superconductivity from Two Alternative Stacked Monolayers in a Bulk 4Hb‐${\mathrm{TaSe}}_{2}$ Heterostructure via Pressure,” Nano Letter 23 (2023): 2121–2128. 10.1021/acs.nanolett.2c04385 36877932

[16] O. J. Clark , M. J. Neat , K. Okawa , et al., “Fermiology and Superconductivity of Topological Surface States in ${\mathrm{PdTe}}_{2}$ ,” Physical Review Letters 120 (2018): 156401. 29756894 10.1103/PhysRevLett.120.156401

[17] J. Cook , S. Mardanya , Q. Lu , et al., “Observation of Gapped Topological Surface States and Isolated Surface Resonances in ${\mathrm{PdTe}}_{2}$ Ultrathin Films,” Nano Letter 23 (2023): 1752–1757. 10.1021/acs.nanolett.2c04511 36825889

[18] M. Z. Hasan and C. L. Kane , “Colloquium: Topological Insulators,” Reviews of Modern Physics 82 (2010): 3045–3067.

[19] B. Bradlyn , J. Cano , Z. Wang , et al., “Beyond Dirac and Weyl fermions: Unconventional Quasiparticles in Conventional Crystals,” Science 353 (2016): aaf5037. 27445310 10.1126/science.aaf5037

[20] S.‐Y. Xu , I. Belopolski , N. Alidoust , et al., “Discovery of a Weyl Fermion Semimetal and Topological Fermi Arcs,” Science 349 (2015): 613–617. 26184916 10.1126/science.aaa9297

[21] B.‐Q. Lv , N. Xu , H.‐M. Weng , et al., “Observation of Weyl Nodes in TaAs,” Nature Physics 11 (2015): 724–727.

[22] Y. Shao , A. N. Rudenko , J. Hu , et al., “Electronic Correlations in Nodal‐Line Semimetals,” Nature Physics 16 (2020): 636–641.

[23] A. P. Schnyder , S. Ryu , A. Furusaki , and A. W. Ludwig , “Classification of Topological Insulators and Superconductors in Three Spatial Dimensions,” Physical Review B 78 (2008): 195125.

[24] S. Nadj‐Perge , I. K. Drozdov , J. Li , et al., “Observation of Majorana Fermions in Ferromagnetic Atomic Chains on a Superconductor,” Science 346 (2014): 602–607. 25278507 10.1126/science.1259327

[25] P. Zhang , R. Noguchi , K. Kuroda , et al., “Observation and control of the weak topological insulator state in ${\mathrm{ZrTe}}_{5}$ ,” Nature Communications 12 (2021): 406. 10.1038/s41467-020-20564-8 PMC7813838 33462222

[26] H. Pi , S. Zhang , Y. Xu , Z. Fang , H. Weng , and Q. Wu , “First Principles Methodology for Studying Magnetotransport in Narrow Gap Semiconductors With ${\mathrm{ZrTe}}_{5}$ Example,” npj Computational Materials 10 (2024): 276.

[27] Z. Liu , S. Zhang , Z. Fang , H. Weng , and Q. Wu , “Combined First‐Principles and Boltzmann Transport Theory Methodology for Studying Magnetotransport in Magnetic Materials,” Physical Review Research 6 (2024): 043185.

[28] J. Hu , F. Yu , A. Luo , X.‐H. Pan , J. Zou , X. Liu , and G. Xu , “Chiral Topological Superconductivity in Superconductor‐Obstructed Atomic Insulator‐Ferromagnetic Insulator Heterostructures,” Physical Review Letters 132 (2024): 036601. 38307042 10.1103/PhysRevLett.132.036601

[29] P.‐F. Liu , F. Zheng , J. Li , et al., “Two‐Gap Superconductivity in a Janus MoSH Monolayer,” Physical Review B 105 (2022): 245420.

[30] P.‐F. Liu , J. Li , C. Zhang , et al., “Type‐II Dirac Cones and Electron‐Phonon Interaction in Monolayer Biphenylene From First‐Principles Calculations,” Physical Review B 104 (2021): 235422.

[31] P.‐F. Liu , J. Li , X.‐H. Tu , et al., “Prediction of Superconductivity and Topological Aspects in Single‐Layer β‐${\mathrm{Bi}}_{2}\mathrm{Pd}$ ,” Physical Review B 102 (2020): 155406.

[32] J. Li , L. Wei , X. Shi , et al., “Machine Learning Accelerated Discovery of Superconducting Two‐Dimensional Janus Transition Metal Sulfhydrates,” Physical Review B 109 (2024): 174516.

[33] N. M. Lakin , G. V. D. Hoek , I. R. Beattie , and J. M. Brown , “The Identification of InOH in the Gas Phase and Determination of Its Geometric Structure,” Journal of Chemical Physics 107 (1997): 4439–4444.

[34] C. J. Bradley and A. P. Cracknell , The Mathematical Theory of Symmetry in Solids: Representation Theory for Point Groups and Space Groups (Oxford University Press, 2009).

[35] Z.‐M. Yu , Z. Zhang , G.‐B. Liu , et al., “Encyclopedia of Emergent Particles in Three‐Dimensional Crystals,” Science Bulletin 67 (2022): 375–380. 36546089 10.1016/j.scib.2021.10.023

[36] T. Han , S. Wang , B. Han , Y. Liu , F. Li , and L. Wang , “Two‐Dimensional Potassium Borides With Hidden Kagome‐Like Lattice: Topological Semimetals, van Hove Singularities, and Superconductivity,” Physical Review B 107 (2023): 235154.

[37] Y. Liu , H.‐J. Zhang , and Y. Yao , “Ab Initio Investigation of Magnetic Transport Properties by Wannier Interpolation,” Physical Review B 79 (2009): 245123.

[38] J. Qiao , J. Zhou , Z. Yuan , and W. Zhao , “Calculation of Intrinsic Spin Hall Conductivity by Wannier Interpolation,” Physical Review B 98 (2018): 214402.

[39] P. Giannozzi , S. Baroni , N. Bonini , et al., “QUANTUM ESPRESSO: A Modular and Open‐Source Software Project for Quantum Simulations of Materials,” Journal of Physics Condensed Matter 21 (2009): 395502. 21832390 10.1088/0953-8984/21/39/395502

[40] P. Giannozzi , O. Andreussi , T. Brumme , et al., “Advanced Capabilities for Materials Modelling With Quantum ESPRESSO,” Journal of Physics Condensed Matter 29 (2017): 465901. 29064822 10.1088/1361-648X/aa8f79

[41] P. Giannozzi , O. Baseggio , P. Bonfà , et al., “Quantum ESPRESSO Toward the Exascale,” Journal of Chemical Physics 152 (2020): 154105. 32321275 10.1063/5.0005082

[42] S. Baroni , S. De Gironcoli , A. Dal Corso , and P. Giannozzi , “Phonons and Related Crystal Properties From Density‐Functional Perturbation Theory,” Reviews of Modern Physics 73 (2001): 515–562.

[43] G. Grimvall , The Electron‐Phonon Interaction in Metals, Vol. 8 (North‐Holland, 1981).

[44] F. Giustino , “Electron‐Phonon Interactions From First Principles,” Reviews of Modern Physics 89 (2017): 015003.

[45] P. B. Allen and R. C. Dynes , “Transition Temperature of Strong‐Coupled Superconductors Reanalyzed,” Physical Review B 12 (1975): 905–922.

[46] W. L. McMillan , “Transition temperature of strong‐coupled superconductors,” Physical Review 167 (1968): 331–344.

[47] D. Liu , W. Zhang , and D. Mou , et al., “Electronic Origin of High‐Temperature Superconductivity in Single‐Layer FeSe Superconductor,” Nature Communications 3 (2012): 931. 10.1038/ncomms1946 22760630

[48] X. Xi , Z. Wang , W. Zhao , et al., “Ising Pairing in Superconducting ${\mathrm{NbSe}}_{2}$ Atomic Layers,” Nature Physics 12 (2016): 139.

[49] Y. Saito , et al., “Superconductivity Protected by Spin–Valley Locking in Ion‐Gated ${\mathrm{MoS}}_{2}$ ,” Nature Physics 12 (2016): 144–149.

[50] Y. Cao , V. Fatemi , S. Fang , et al., “Unconventional Superconductivity in Magic‐Angle Graphene Superlattices,” Nature 556 (2018): 43–50. 29512651 10.1038/nature26160

[51] S. Qin , J. Kim , Q. Niu , and C. K. Shih , “Superconductivity at the Two‐Dimensional Limit,” Science 324 (2009): 1314–1317. 19407146 10.1126/science.1170775

[52] R. Cao , Q.‐L. Yang , H.‐X. Deng , S.‐H. Wei , J. Robertson , and J.‐W. Luo , “Softening of the Optical Phonon by Reduced Interatomic Bonding Strength Without Depolarization,” Nature 634 (2024): 1080–1085. 39478211 10.1038/s41586-024-08099-0

[53] J. P. Perdew , K. Burke , and M. Ernzerhof , “Generalized Gradient Approximation Made Simple,” Physical Review Letters 77 (1996): 3865–3868. 10062328 10.1103/PhysRevLett.77.3865

[54] G. Kresse and J. Furthmüller , “Efficient Iterative Schemes for Ab Initio Total‐Energy Calculations Using a Plane‐Wave Basis Set,” Physical Review B 54 (1996): 11169–11186. 10.1103/physrevb.54.11169 9984901

[55] Q.‐S. Wu , S.‐N. Zhang , H.‐F. Song , M. Troyer , and A. A. Soluyanov , “WannierTools: An Open‐Source Software Package for Novel Topological Materials,” Computer Physics Communications 224 (2018): 405–416.

[56] A. A. Mostofi , J. R. Yates , G. Pizzi , et al., “An updated version of wannier90: A Tool for Obtaining Maximally‐Localised Wannier Functions,” Computer Physics Communications 185 (2014): 2309–2310.

[57] J. Gao , Q.‐S. Wu , C. Persson , and Z. Wang , “Irvsp: To Obtain Irreducible Representations of Electronic States in the VASP,” Computer Physics Communications 261 (2021): 107760.

