# Supplementary material for: Type‐II Dirac Fermions in Monolayer In2O: Interplay of Magnetotransport, Spin Hall Effect, and Superconductivity
Source: Adv Sci (Weinh). 2026 Apr 13:e24346. Online ahead of print. doi: 10.1002/advs.202524346 (PMC13334878; doi:10.1002/advs.202524346)
Supplement: Supplementary file 1 — Supporting File: advs75194‐sup‐0001‐SuppMat.pdf. [file ADVS-9999-e24346-s001.pdf]

Supplemental Information of "Type-II Dirac Fermions in 2D Materials Interplay of Magnetotransport, Spin Hall Effect, and Superconductivity"

Qing-Bo Liu,[1] De-Ming Feng,[1] Wen-Neng Zhao,[1] Yu-Yang Zhao,[1] Lun Xiong,[1,*] Xing-Yi Tan,[2,3,†] and Hua-Hua Fu[4,5,‡]

[1]*Hubei Key Laboratory of Optical Information and Pattern Recognition, School of Optical Information and Energy Engineering, School of Mathematics and Physics, Wuhan Institute of Technology, Wuhan, 430073, China*

[2]*Department of Physics, Chongqing Three Gorges University, Wanzhou, 404100, China*

[3]*College of Intelligent systems science and engineering, Hubei Minzu University, Enshi, 445000, China*

[4]*School of Physics and Wuhan National High Magnetic Field Center, Huazhong University of Science and Technology, Wuhan 430074, People's Republic of China.*

[5]*Institute for Quantum Science and Engineering, Huazhong University of Science and Technology, Wuhan, Hubei 430074, China.*

(Dated: March 15, 2026)

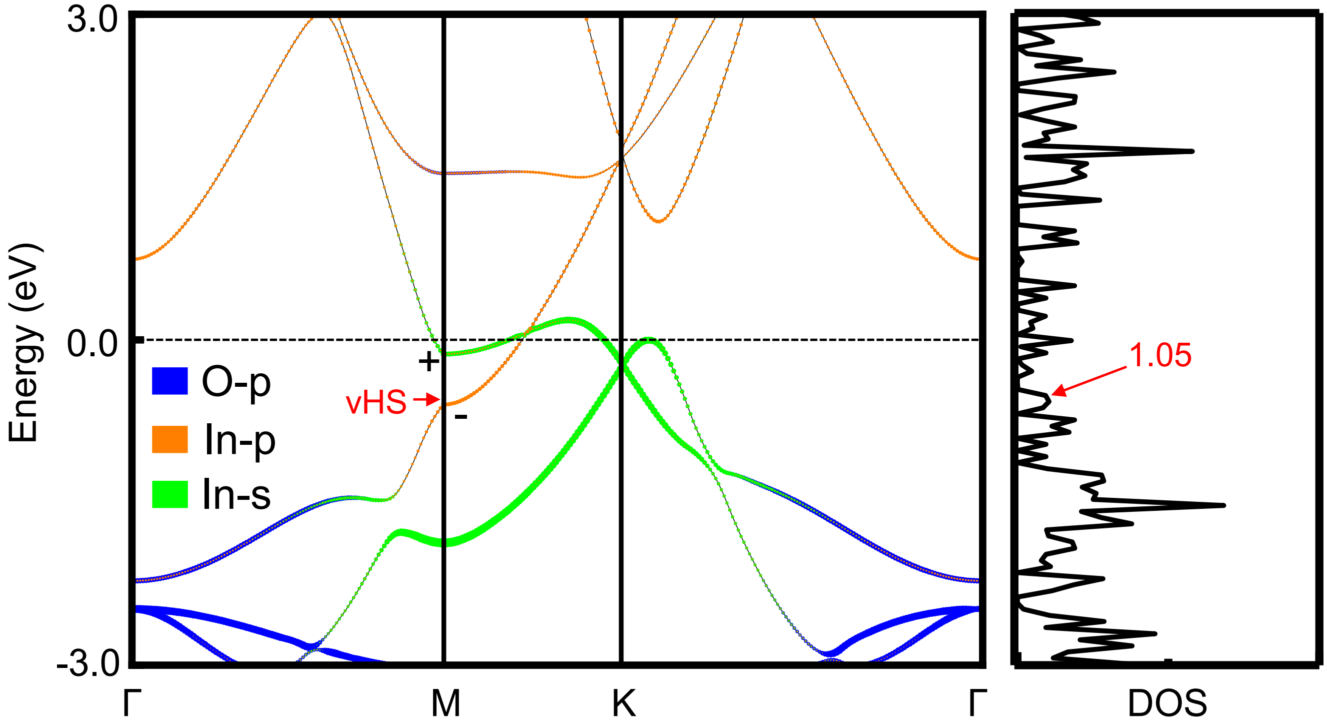

FIG. S1. The projected band structures and DOS of In_2O without SOC.

We notice that the In-4p orbitals and In-4s orbitals are very extended, which also exhibits considerable amplitude under the type-II Dirac points near E_F, implying a band inversion between the In-4p and In-4s states. The character is further verified by the projected band structures in Fig. 1, which demonstrates that the In-4s states with even parity are lower by 0.55 eV than the In-4p states with odd parity at the M point. It can form the Van Hove singularities (VHSs) with a DOS peak (1.05) at the M point in Fig. S1. Molecular dynamics simulations reveal that the monolayer 5×5×1 In_2O supercell remains structurally stable at temperatures up to 300 K but undergoes structural collapse at 500 K, thereby demonstrating its stability at room temperature in Fig. S2. Furthermore, calculations of the Berry curvature show that the curvature induced by type-I Weyl points is notably stronger than that from type-II Weyl points, resulting in a peak in the spin Hall conductivity at -0.31 eV. We found that when a 0.5 eV/Å electric field is applied along the z-direction to 2D In2O, the space inversion symmetry is broken, leading to the destruction of both type-II and type-I Dirac points in Figure S4(a). Additionally, the spin Hall conductance increases from a maximum of 125 to 329.6 Ω/cm in Figure S4(b). .

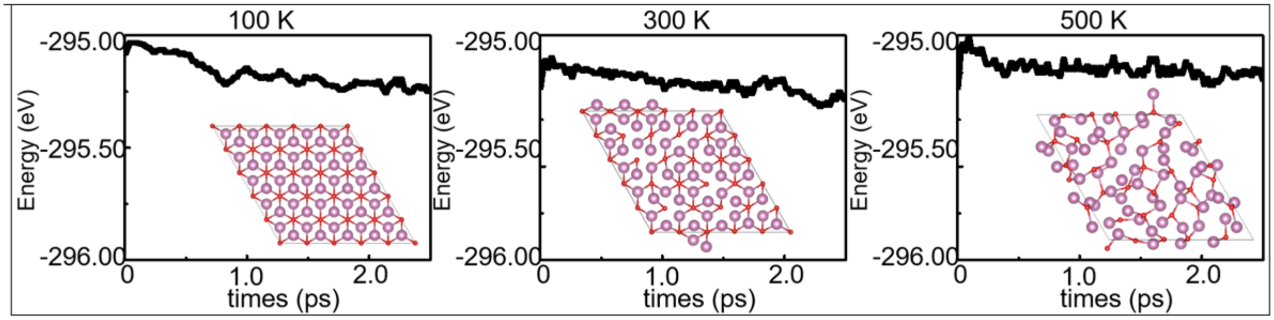

FIG. S2. The ab initio molecular dynamics simulation in 100, 300, and 500 K with 5*5*1 spuercells.

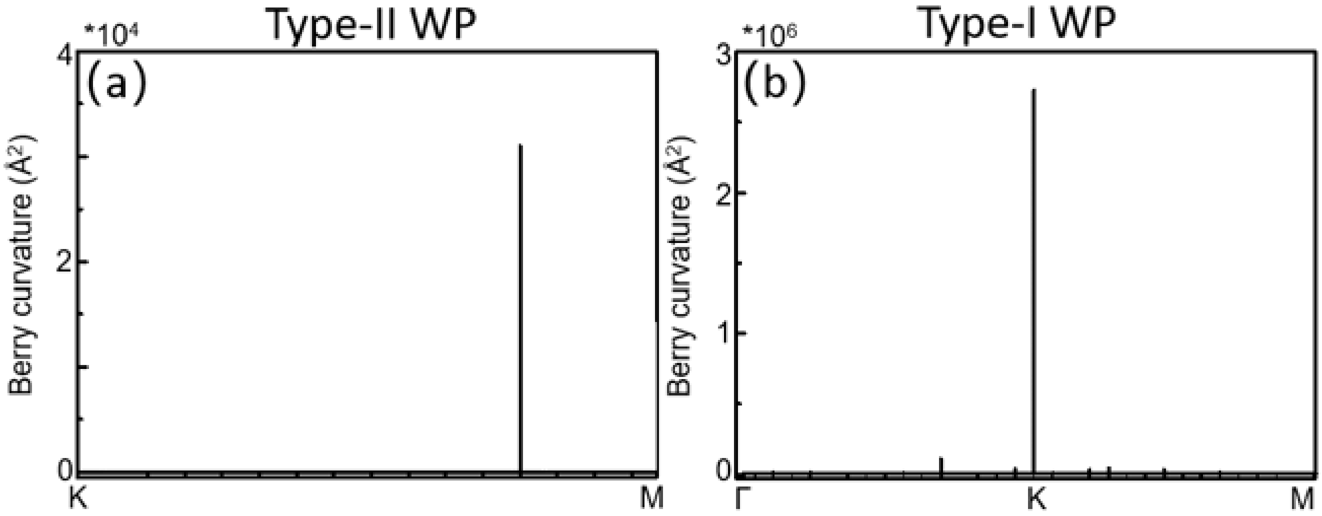

FIG. S3. Berry curvature distribution of type-I and type-II Weyls.

* xionglun@wit.edu.cn
† tanxy@sanxiau.edu.cn
‡ hhfu@hust.edu.cn

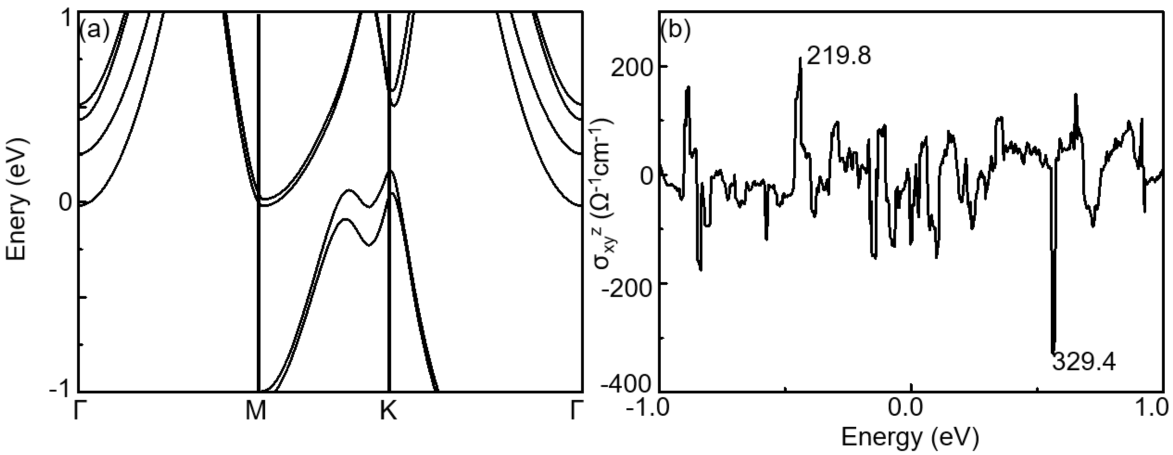

FIG. S4. (a) It shows the SOC band after applying a 0.5 eV/Å electric field along the z-direction, and (b) shows the spin Hall conductance after applying a 0.5 eV/Å electric field.
